# Supplementary material for: Indene-Derived Hydrazides Targeting Acetylcholinesterase Enzyme in Alzheimer’s: Design, Synthesis, and Biological Evaluation
Source: Pharmaceutics. 2022 Dec 28;15(1):94. doi: 10.3390/pharmaceutics15010094 (PMC9860787; doi:10.3390/pharmaceutics15010094)
Supplement: Supplementary file 1 [file pharmaceutics-15-00094-s001.zip › pharmaceutics-2043479-supplementary.pdf]

Indene-derived hydrazides targeting Acetylcholinesterase enzyme in Alzheimer: Design, Synthesis, and Biological Evaluation

Shraddha Manish Gupta [1,2], Ashok Behera [3], Neetesh K Jain [1], Devendra Kumar [3], Avanish Tripathi [4], Shailesh Mani Tripathi [5], Somdutt Mujwar [6], Jeevan Patra [2], Arvind Negi [7,*]

[1] Faculty of Pharmacy, Oriental University, Indore-453555, Madhya Pradesh, India; Shraddha.27981@gmail.com (S.M.G); drneetesh@orientaluniversity.in (N.K.J)

[2] Department of Pharmaceutical Sciences, School of Health Sciences and Technology, University of Petroleum and Energy Studies (UPES), Dehradun- 248007, India; jeevanpatra96@gmail.com (J.P.)

[3] Faculty of Pharmacy, DIT University, Dehradun-248009, Uttarakhand, India; ashokiicb2015@gmail.com (A.B.); devendrak.phe@gmail.com (D.K.)

[4] Institute of Pharmaceutical Research, GLA University, Mathura-281 406 (U.P.), India; avanish.rs.phe14@iitbhu.ac.in (A.T.)

[5] Department of Pharmacy, Birla Institute of Technology and Science, Pilani Campus, Rajasthan, 333031, India; manishailesh08@gmail.com (M.T.)

[6] Chitkara College of Pharmacy, Chitkara University, Rajpura-140401 Punjab, India; somduttmujwar@gmail.com (S.M.)

[7] Department of Bioproduct and Biosystems, Aalto University, FI-00076 Espoo, Finland; arvind.negi@aalto.fi (A.N.)

* Correspondence: arvind.negi@aalto.fi

^{1}H NMR Spectra

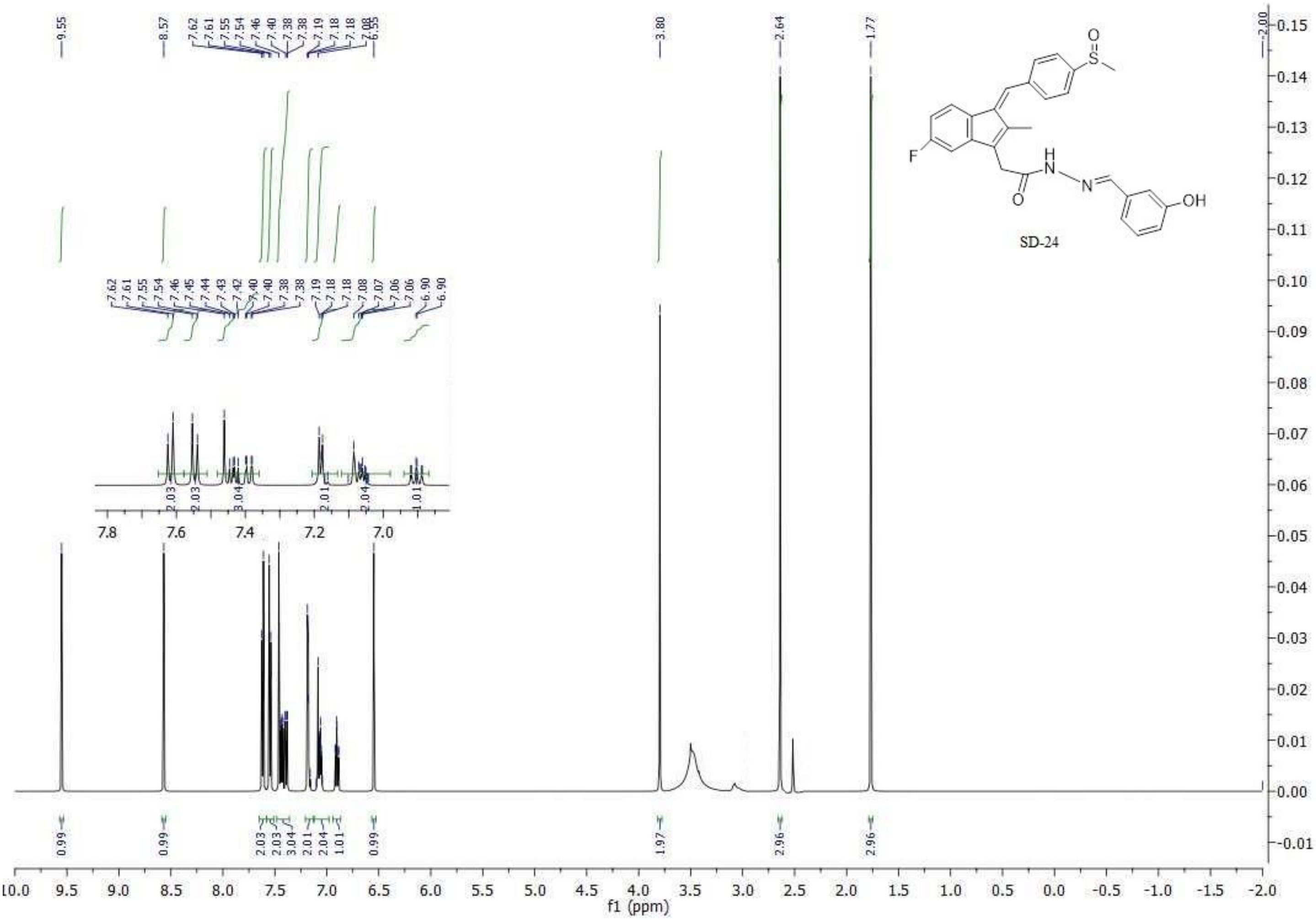

Figure S1: ^{1}H NMR Spectra of SD-24

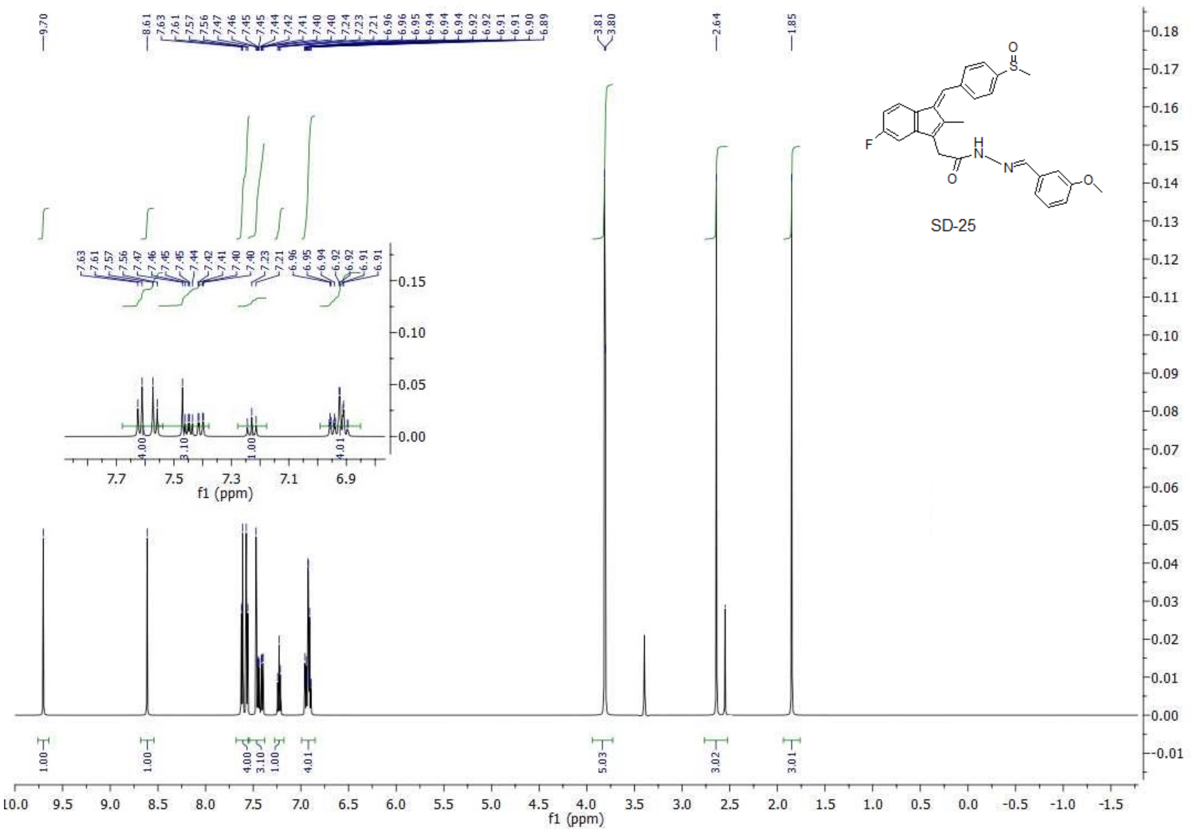

Figure S2: ^{1}H NMR Spectra of SD-25

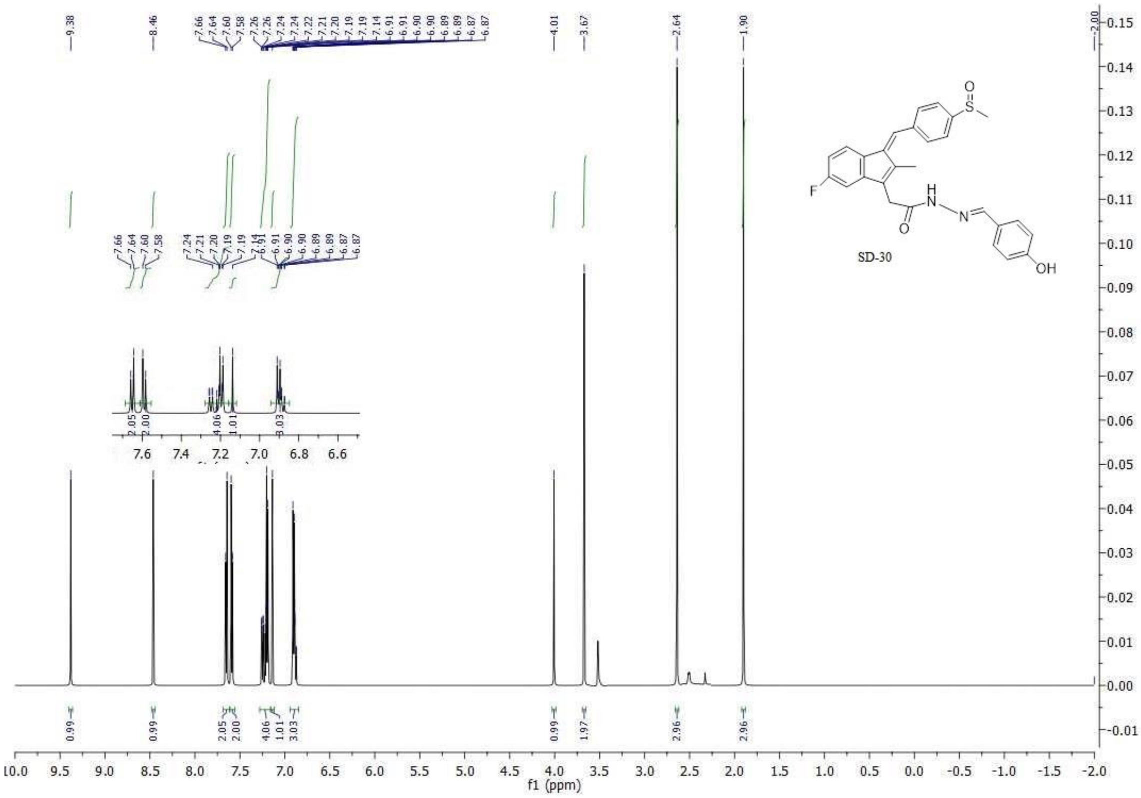

Figure S3: ^{1}H NMR Spectra of SD-30

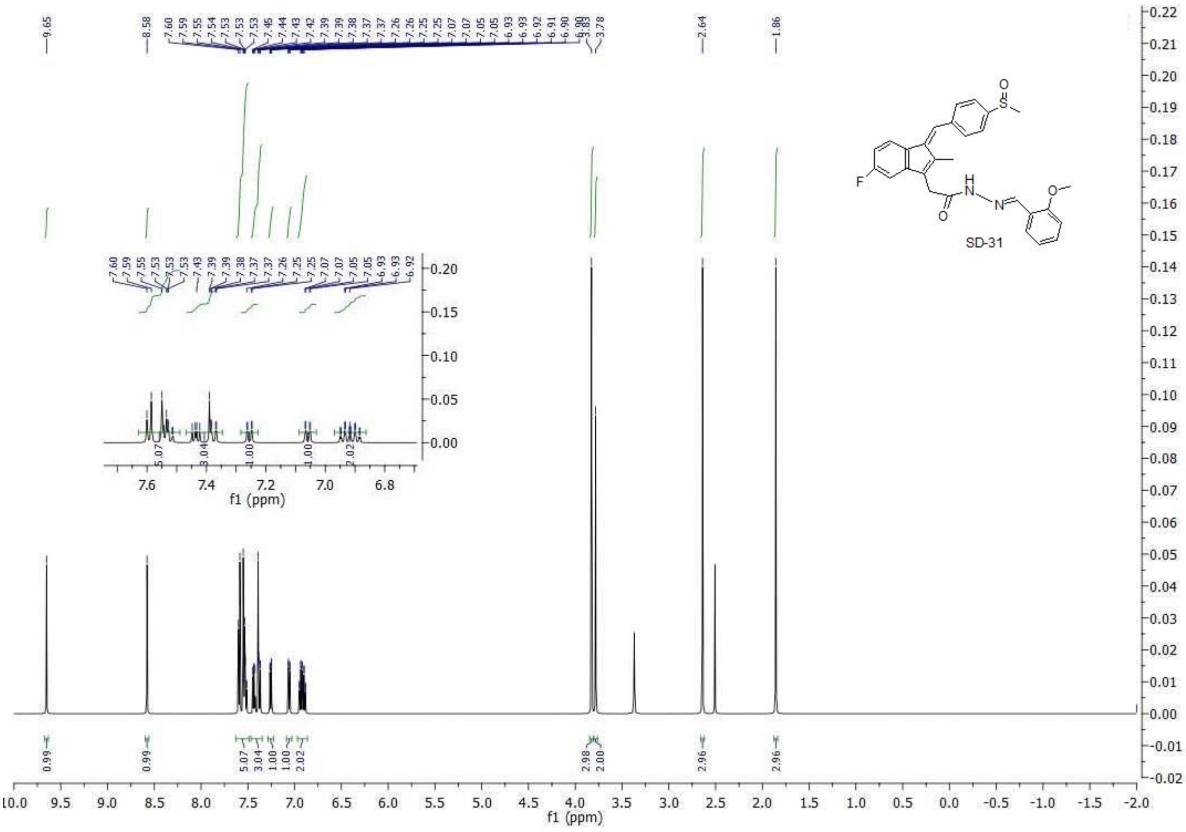

Figure S4: ^{1}H NMR Spectra of SD-31

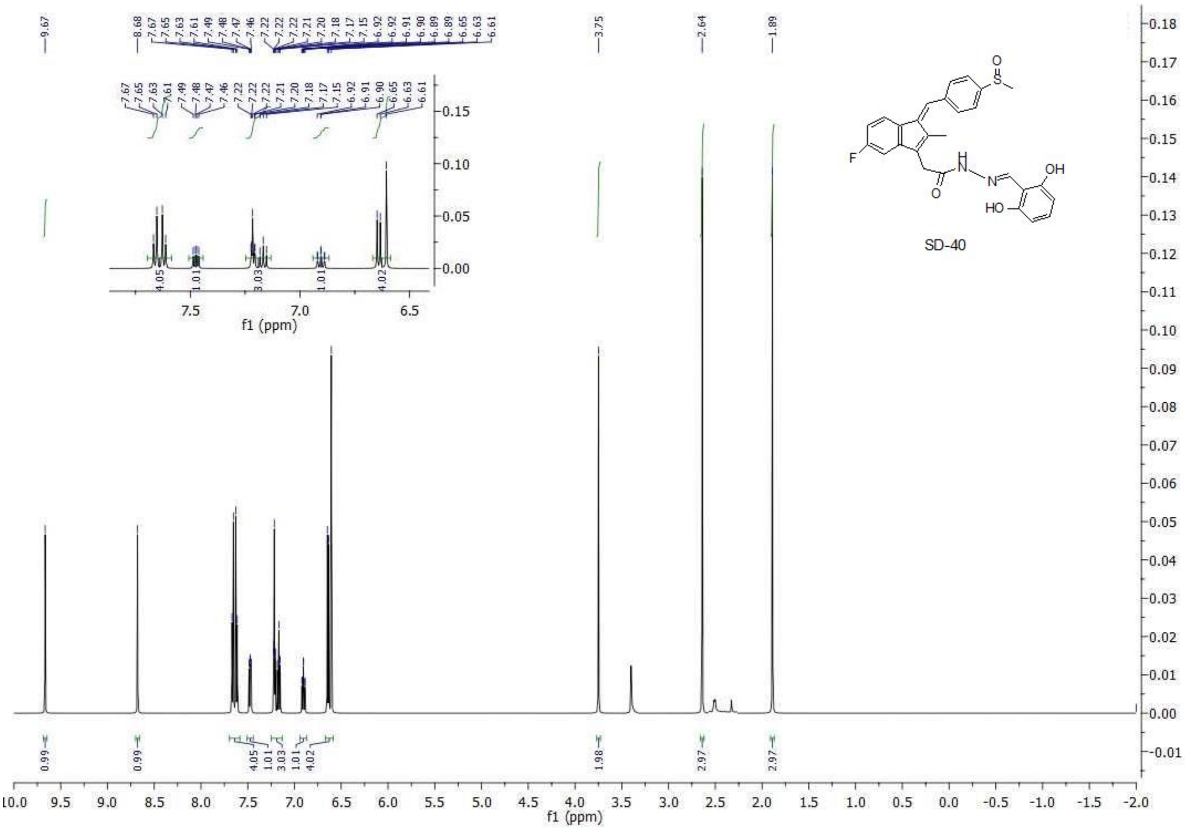

Figure S5: ^{1}H NMR Spectra of SD-40

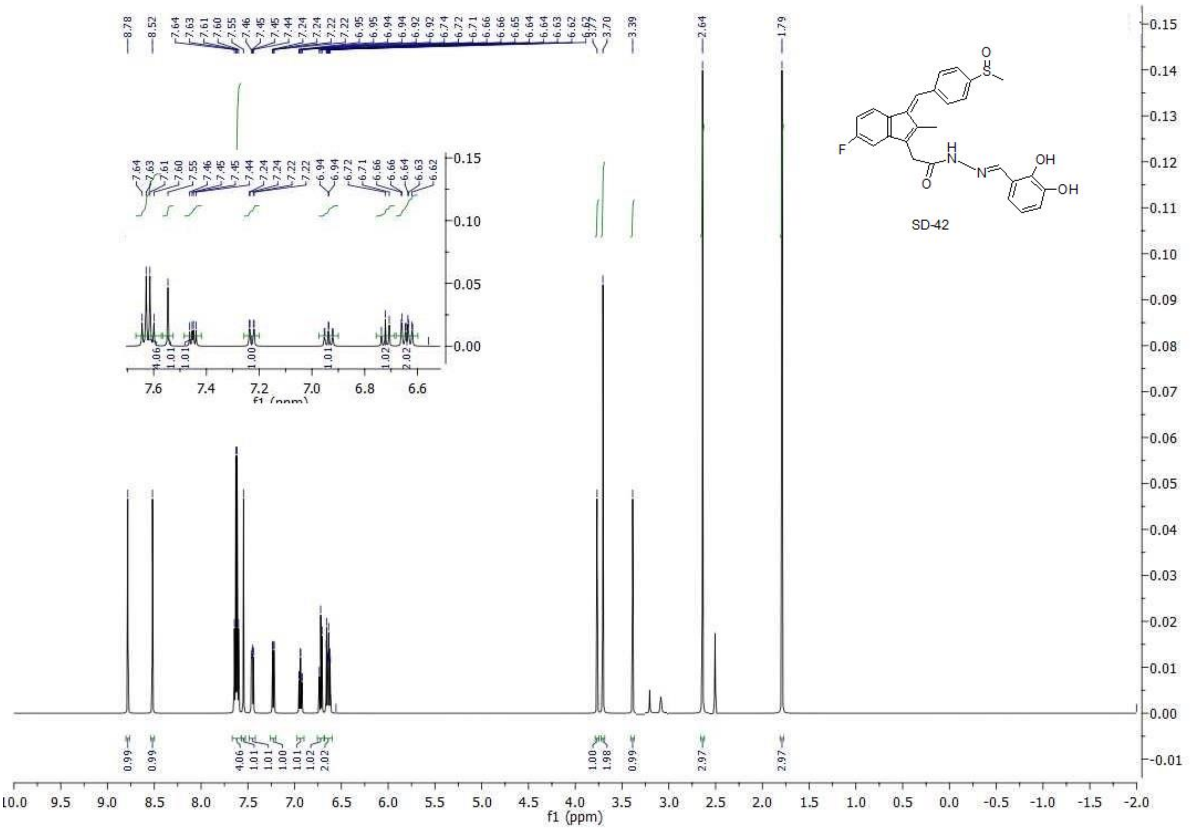

Figure S6: ^{1}H NMR Spectra of SD-42

^{13}C NMR Spectra

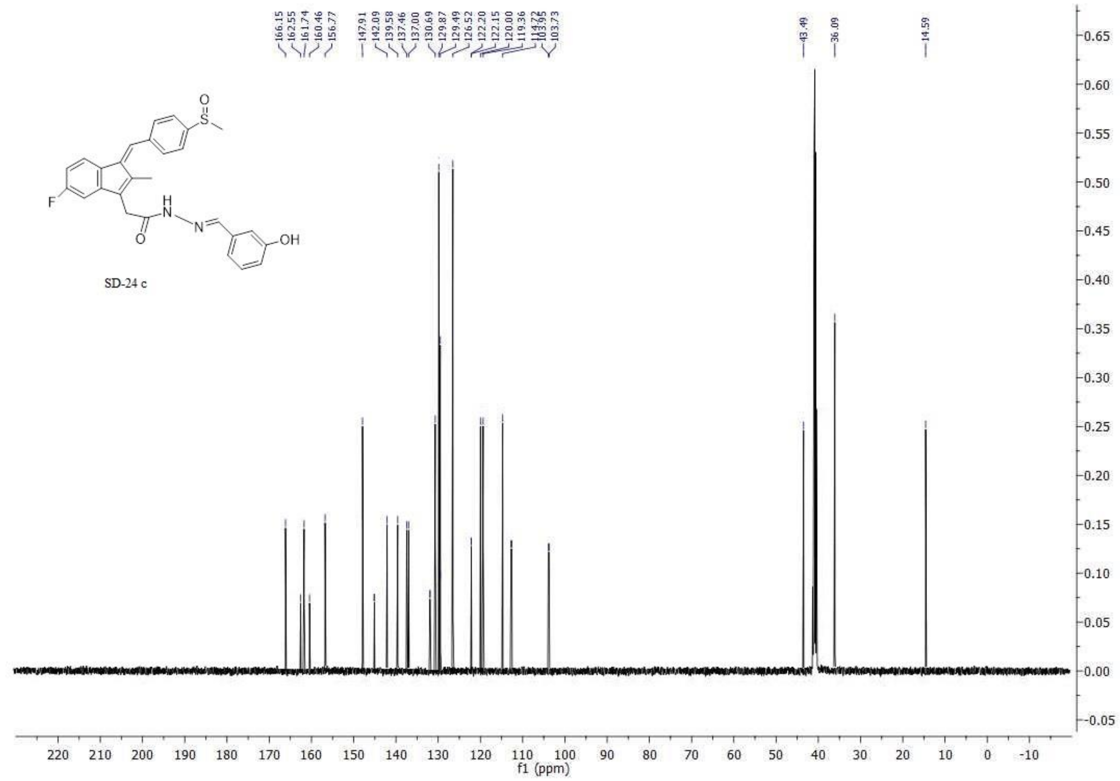

Figure S7: ^{13}C NMR Spectra of SD-24

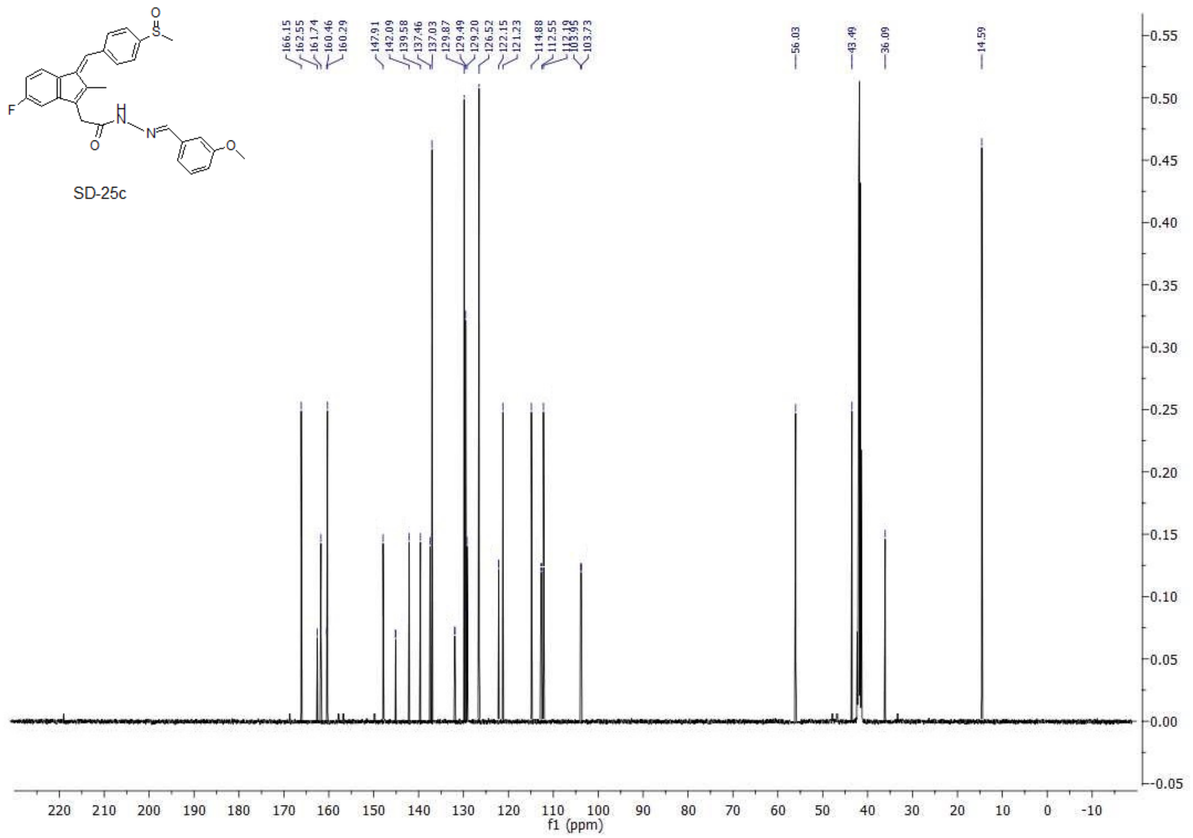

Figure S8: ^{13}C NMR Spectra of SD-25

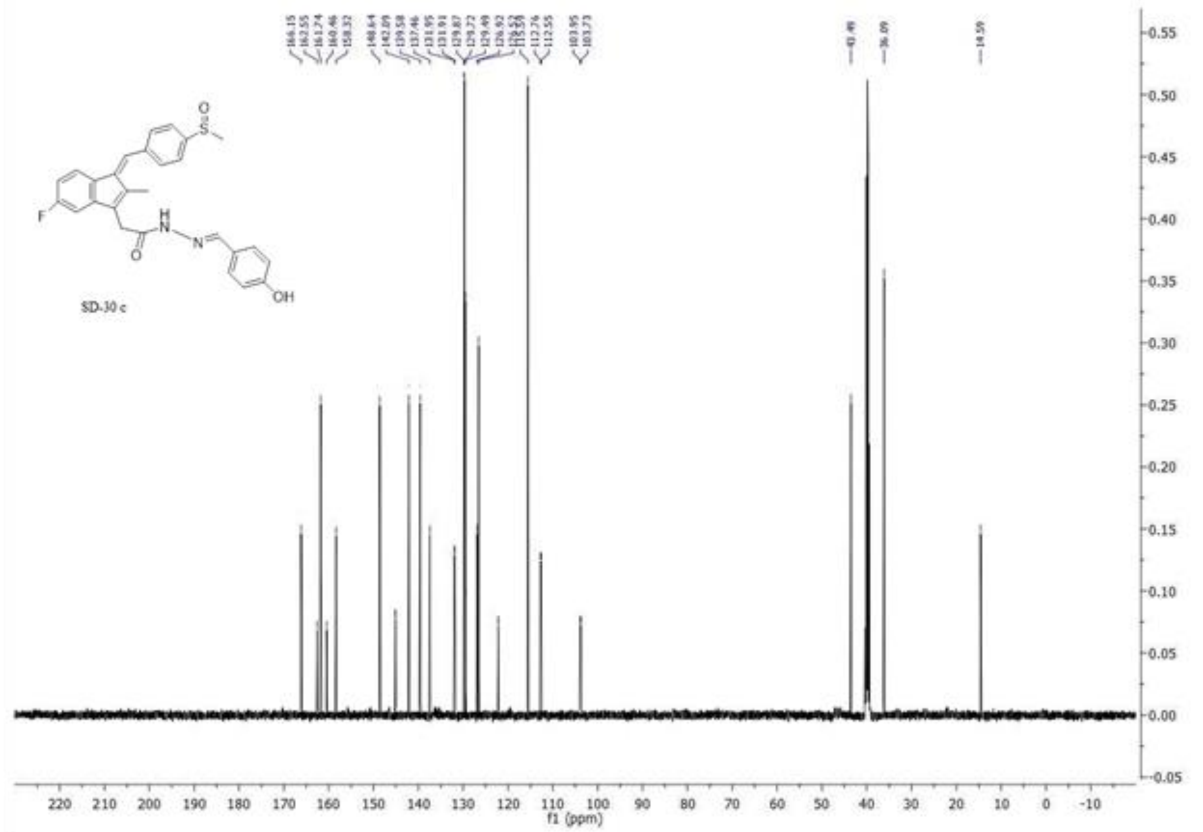

Figure S9: ^{13}C NMR Spectra of SD-30

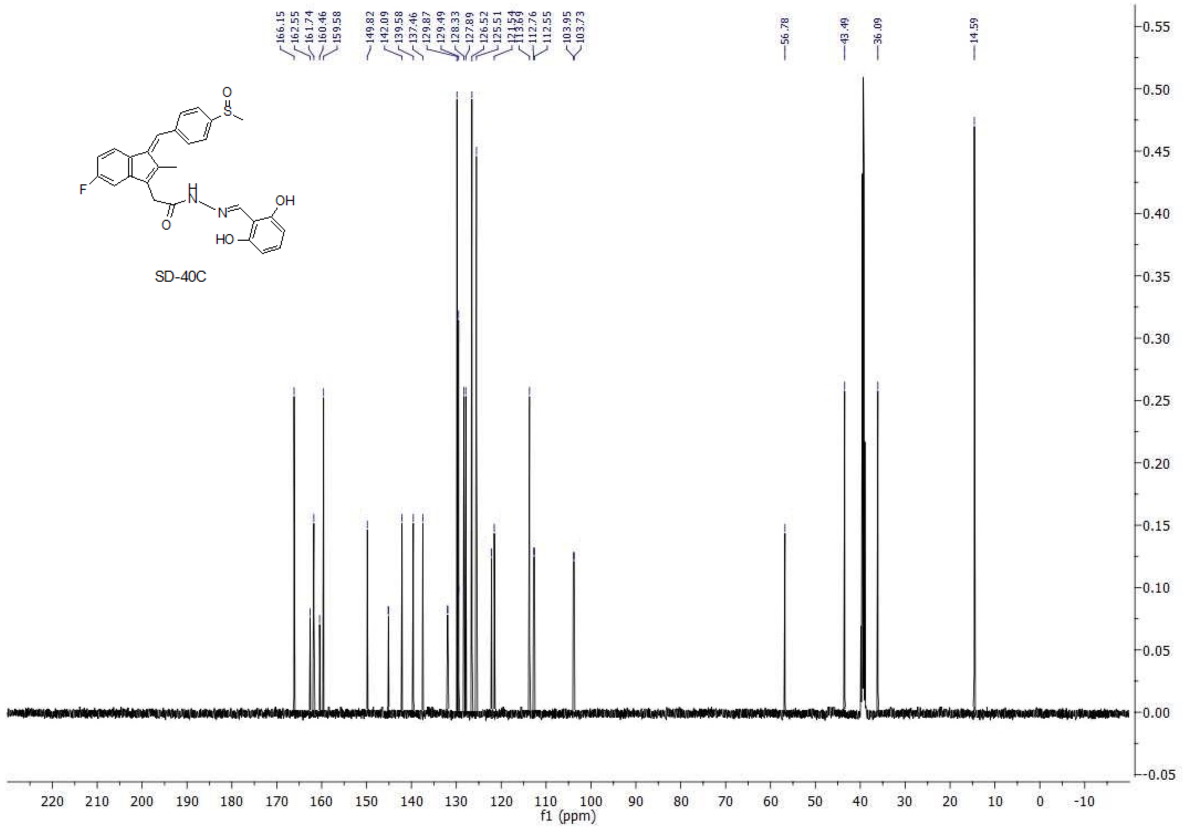

Figure S10: ^{13}C NMR Spectra of SD-40

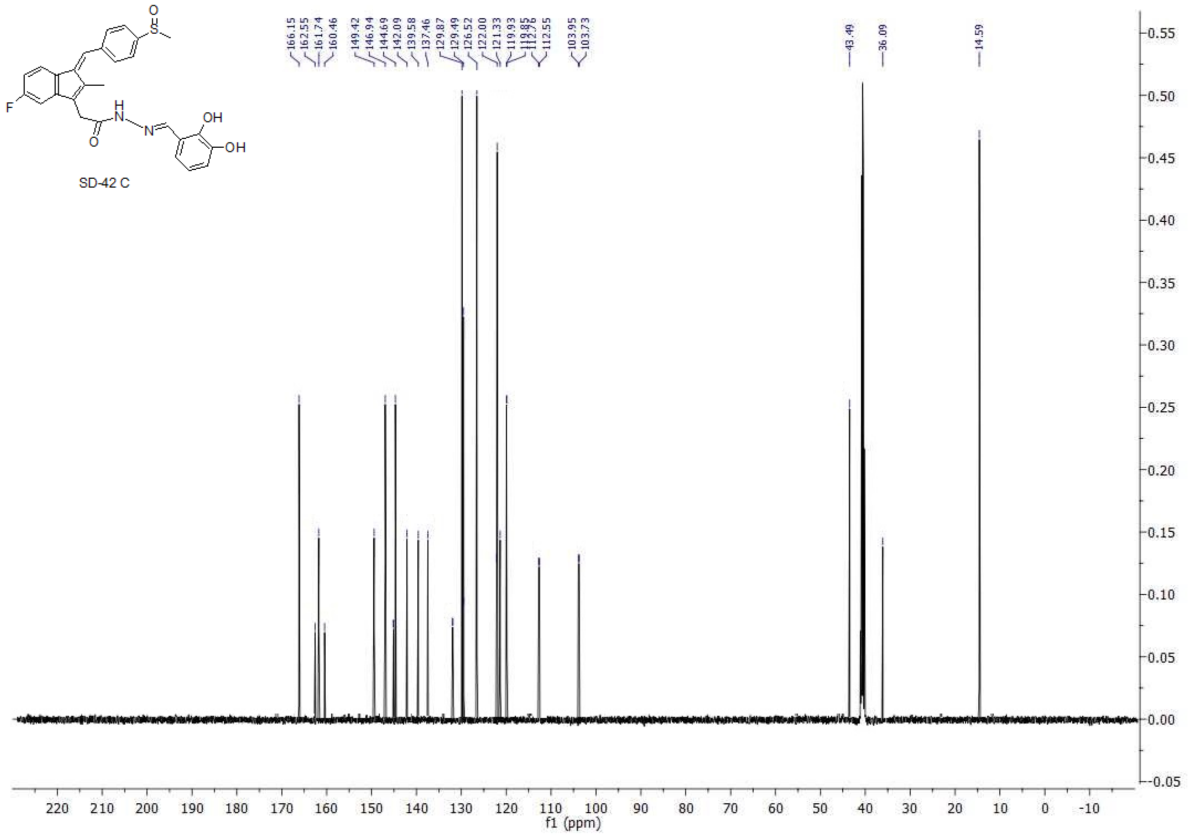

Figure S11: ^{13}C NMR Spectra of SD-42

Mass Spectra

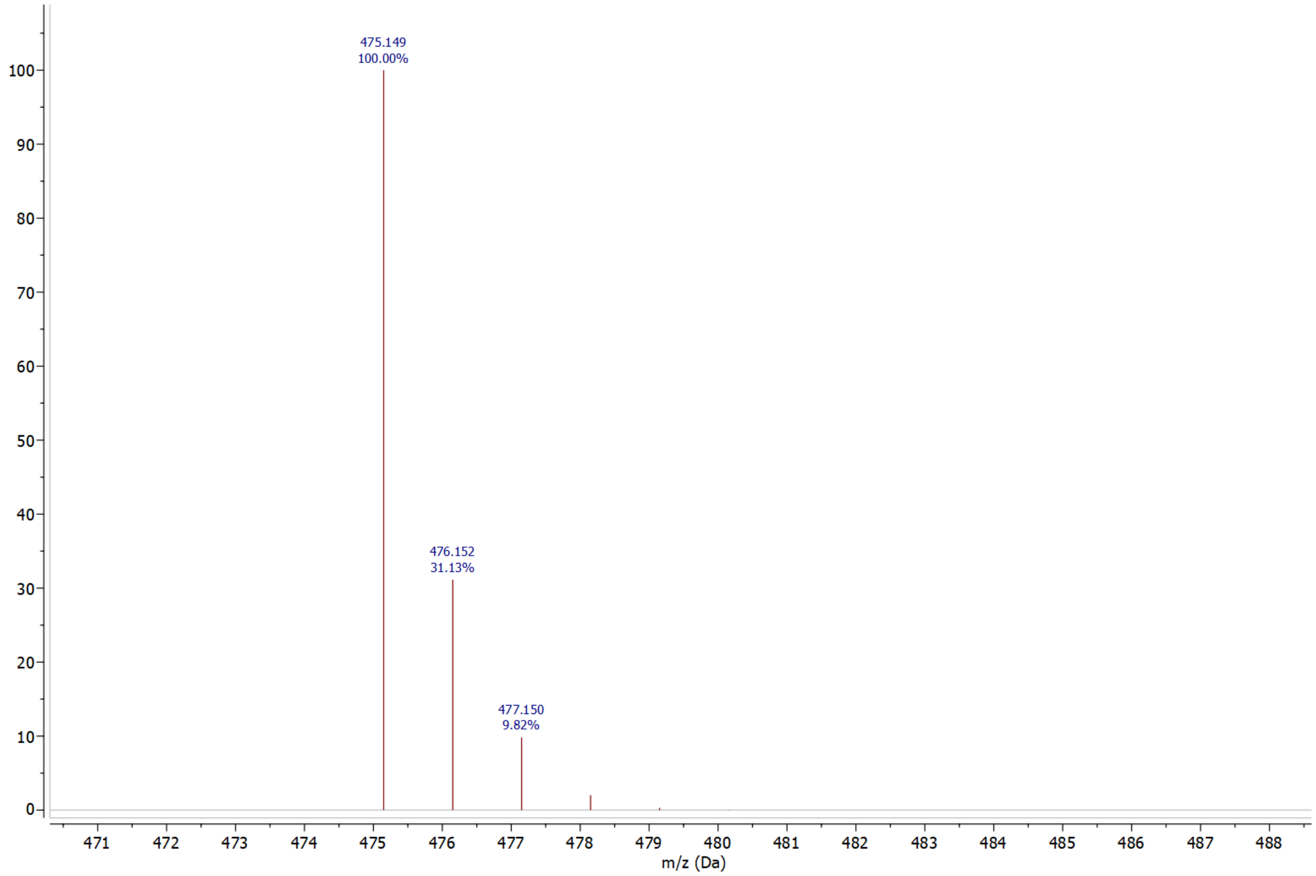

Figure S12: Mass Spectra of SD-24
